# Factors associated with scabies severity and reinfection: A cross-sectional study during recent surges in the Chattogram Division, Bangladesh

**DOI:** 10.1016/j.ijregi.2026.100899

**Published:** 2026-04-15

**Authors:** Md. Safayet Hossain, Dulal Chandra Nandi, Puja Sree Biswas, Rakibul Islam, Sosunta Saha, Md Monirul Islam

**Affiliations:** 1 Department of Statistics, Comilla University, Cumilla-3506, Bangladesh; 2 Global Public Health Research Foundation, Dhaka-1230, Bangladesh

**Keywords:** Scabies, Severity, Reinfection, Treatment effectiveness, Heatmap, Bangladesh

## Abstract

**Objectives:**

This study aimed to assess the severity level, reinfection pattern, and associated factors of scabies in the Chattogram Division of Bangladesh.

**Methods:**

This is a cross-sectional household-based study conducted among 349 scabies-affected participants using multi-stage cluster sampling. Data were collected through face-to-face interviews using a structured questionnaire. The study considered two primary outcome variables, namely, severity and reinfection rate. Descriptive analyses were conducted to show frequency and percentage, heatmapping for geographical variation, and chi-square tests for bivariate association; to identify the associated factors, multinomial logistic regression was performed.

**Results:**

Of the participants, 44.3% experienced severe scabies, whereas 34.1% of the participants faced reinfection three or more times in the last 12 months. Chattogram district showed the highest hotspot burden for severity and reinfection. Children and older adults had significantly higher odds of severe scabies problems. Low educational attainment, infrequent bathing habits, pus discharge, poor hygiene, excessive dust, sharing bed or cloth items, and family history of scabies or skin disease were strongly associated with increasing severity and reinfection. In addition, combined treatments are found to be more effective than traditional methods or single treatments.

**Conclusion:**

This study reveals a significant burden of scabies severity and reinfection that emphasizes household-focused and community-based treatments.

## Highlights

• Severe scabies was found in 44.3% of participants in Chattogram.
• Reinfection was common, with 34.1% reporting three or more episodes in the last year.
• Chattogram district was identified as a hotspot for severity and reinfection.
• Children, older adults, and low-educated groups had higher severity and reinfection.
• Poor hygiene and household transmission increased risk; combined treatment worked best.

## Introduction

Skin diseases and their complications significantly impact our quality of life [1,2]. Scabies is a highly contagious ectoparasitic skin infestation caused by the mite Sarcoptes scabiei var. hominis, is a major public health concern, particularly, in places with a high population density and inadequate facilities [3,4]. It has been largely ignored tropical disease that is mostly widespread in lower- and middle-income South East Asian nations [5]. It is a common skin condition that affects people of all ages, races, and socioeconomic situations worldwide [6]. Poor hygiene, overcrowding, and a limited health care infrastructure deteriorate the condition. Scabies is known to cause severe sores, intense itching, especially at night, along with skin lesions, and, in the worst cases, abscesses and bacterial infections [7,8].

Scabies is still a major dermatological and community health issue in Bangladesh [9]. It exists as a major public health issue in urban and rural environments due to social and economic challenges, as well as densely living conditions, which enable continued transmission of the disease [10]. Infestation rates of scabies have been profoundly high among children and refugee populations. The spread of scabies is influenced by poor personal hygiene, shared bedding or clothing, and prolonged close contact [11]. Although the World Health Organization classified scabies as a neglected tropical disease in 2017, the real burden of the disease remains inadequately understood, especially in rural areas of Bangladesh [12]. This lack of knowledge seems to be prevalent among poor communities, which reduces the efficacy of control of the disease. The difficulty of stopping the life cycle of the mite and the transmission throughout families and communities makes reinfection a major barrier to scabies management [13]. However, initial treatment reduces prevalence, failure to address environmental reservoirs, or close relationships might result in reinfection of the disease [14].

Several studies have found high prevalence among specific populations. For example, high rates were found in several Madrasahs in Dhaka, with 34% affected by scabies. Male students, younger age, sharing of bedding, close contacts, and a history of pruritus were the risk factors associated with scabies [10]. Similarly, the prevalence of scabies in Rohingya refugees living in Cox’s Bazar camps was reported to be as high as 66%. Contributing factors associated with higher infection rates include having dirt floors, contact with domestic and stray animals, dust, and cold weather [15]. In addition, over the course of 16 months, more than 178,000 consultations for scabies occurred in the camps [3]. These findings demonstrate the scope of the outbreak and the ongoing difficulties in preventing reinfection and ensuring adequate treatment coverage.

Although several studies have addressed the prevalence and associated risk factors, there are not much data available at the district level prevalence, severity, and reinfection with scabies in community settings, especially in the Chattogram Division, which has a harmonious blend of rural and urban populations. Along with these issues, late diagnosis, lack of health awareness, and poor understanding of treatment management also cause barriers to effective control. In addition, not much is known about the factors that influence scabies treatment-seeking behavior. Identifying these gaps require designing focused methods and close observation, especially in marginalized areas that discourage treatment-seeking behavior.

This research aims to fill these gaps by estimating the scabies severity, reinfection rates, and treatment effectiveness of scabies in the Chattogram Division. It also seeks to identify the socioeconomic and environmental factors that are associated with the outcome variables. In addition, this study wants to observe effective treatment method for scabies that participants rely on.

## Methods and materials

### Study design and settings

This study is a cross-sectional study. It is conducted in selected districts of the Chattogram Division. The study used a multi-stage cluster sampling design with household-based systematic and consecutive participant selection. At the first stage, Chattogram Division was purposively selected as the primary sampling unit from the eight administrative divisions of Bangladesh due to its large population size and socio-demographic diversity. At the second stage, five districts (Chattogram, Cumilla, Chandpur, Noakhali, and Brahmanbaria) are selected out of the 11 districts of Chattogram Division using simple random sampling. We have purposively excluded the two hilly districts, Rangamati and Bandarban, due to low population density (Figure S1). At the third stage, the sample size for each selected district was calculated using Cochran’s formula for estimating population proportions, assuming an unknown prevalence of scabies (*P* = 0.5) at a margin of error = 7-13% and a 95% confidence level. The total sample was then distributed across districts using probability proportional to size based on district population. In the end, a systematic household-based strategy was used for data collection because the whole household-level sampling frame was not accessible. Within each chosen cluster, a beginning point was chosen at random, and households were visited on a regular basis.

### Questionnaire development and data collection

Data were collected from 349 participants using a structured and pre-tested questionnaire administered in English and then read out to the participants in Bengali by the interviewers in face-to-face interviews. The questionnaire was developed after a comprehensive review of relevant literature and adapted from previously validated instruments used in global settings. The validity of the questionnaire was confirmed through expert assessment and pilot testing. To ensure the quality of the instrument, it was reviewed by one dermatologist and one public health specialist who assessed each item for clarity, relevance, and appropriateness. A pilot survey was then conducted with 30 participants to identify minor issues, and some wordings were adjusted to improve pertinence. Together, these steps ensured that the questionnaire was clear, contextually appropriate, and valid for assessing association between potential factors and disease severity and reinfection rates among the study population.

### Inclusion and exclusion criteria

The participants were recruited based on the clinical signs of scabies, according to the criteria set by the International Alliance for the Control of Scabies (IACS). Participants were selected based on clinical signs of scabies, and 85.1% had a physician-confirmed diagnosis, whereas 14.9% were identified based on typical clinical features. This approach is consistent with the clinical (level B) and suspected (level C) categories of the IACS, which included the presence of common skin lesions like papules, pustules, and burrows; the distribution of the lesions, which include the hands, wrists, web of the fingers, abdomen, and genital area; and the history of the lesions, which include itching and close contacts with people infected with scabies.

### Variables of interest

#### Outcome variables

The severity of scabies infection and the reinfection rate among the affected individuals were taken into consideration as outcome variables in this study. Severity was classified into mild, moderate, and severe, depending on the number of body parts involved and the presence of discharge. Mild infection was considered to be the visible scabies symptoms involving less than three body parts and participants who were in the recovery stage. Moderate infection was the involvement of three or more body parts without discharge, whereas severe infection was the involvement of three or more body parts along with discharge. In addition, the reinfection rate was classified depending on the number of times an individual was reinfected with scabies in the last 12 months, which included once, twice, thrice, and four times or more.

#### Covariates

To identify the factors associated with severity and reinfection rate, a range of covariates were considered. These covariates were grouped into seven broad categories, namely, socio-demographic variables (age, gender, education, marital status, district, occupation, religion, monthly income, and number of household members), environmental factors (floor type, residence, dust exposer, sleeping place, use of mosquito net, contact with animals), hygiene and living conditions (bathing frequency, soap use, nail cutting frequency, shared items, shared room, self-rated hygiene), exposure, and contact history (scabies before COVID-19 and after COVID-19, number of reinfections, family history of scabies and other skin diseases), clinical symptoms and diagnosis (current itching, visible rash, push discharge, doctor diagnosis, and severity), and treatment and recovery. Moreover, treatment-related factors were included, such as treatment type, place of treatment, improvement after treatment, and effectiveness of the treatment.

### Statistical analysis

We carried out data extraction, and the results were descriptively provided for response patterns for each variable individually. The percentage was used to show the frequency and prevalence of the categorical variables. For the graphical representation, the visualization techniques included heat mapping for the selected districts of the Chattogram Division. It was also used to show the spatial distribution of scabies severity and reinfection rate in the selected areas. For bivariate analysis, chi-square tests were applied to evaluate the association between covariates and the outcome variables. The multinomial logistic regression was used to identify the associated factors related to reinfection rate and severity of the scabies.

### Evaluation and validation criteria

A reliability test was performed to assess the responses’ reliability; the results showed sufficient internal consistency among the items, with a Cronbach α score of 0.732. Q-Q plot ensures the normality of the data. Multicollinearity was examined using variance inflation factors, all of them were less than 5, indicating that there were no significant problems with multicollinearity among the independent variables.

## Results and findings

### Socio-demographic and disease-related characteristics of the participants

**Figure 1:**
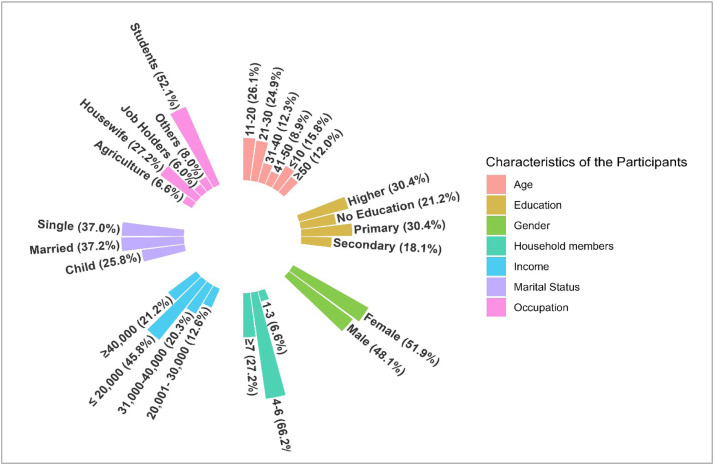
Socio-demographic characteristics of the participants.

Figure 1 shows the socioeconomic characteristics of the respondents. The majority of the participants belong to the age groups 11-20 years (26.07%) and 21-30 years (24.93%), whereas only 12% of the respondents are aged older than 50 years. The gender ratio shows almost equal numbers, the highest number of respondents were students (52.15%), and 27.22% were housewives. Approximately half of the respondents (45.85%) earn less than 20,000 BDT per month, and only 21.20% earn more than 40,000 BDT. Figure S2 presents the distribution of environmental factors among the participants. A significant proportion of the respondents had a cemented floor type. About 79.49% of the respondents lived in buildings, 21.78% had tin shed houses, and 7.74% had mud houses with 18.05% severe dust exposure. Our findings also indicate that 83.09% of the participants’ sleeping place is a bed, whereas others sleep on the floor. In addition, we found that 30.66% have close contact with pets or street animals.

Figure S3 illustrates the hygiene and living conditions of the participants, showing that 13.75% do not frequently bathe, 8.88% do not use soap while bathing, and 14.33% do not frequently cut their nails. Another finding to consider is that 80.23% share their bedding items, 13.75% share their toiletries, and 6.02% share their clothes. Furthermore, only 8.02% claim that they have excellent hygiene, 61.32% have good hygiene, and 30.66% have poor hygiene. Figure S4 depicts the treatment and recovery of the participants, such that 90.8% sought medical help. Among them, 79.01% had formal treatment, 21% had informal treatment, and 96.89% were prescribed medication. Regarding treatment modalities, 29.9% used ointment, 14.3% took medicine, 49.15% used both, and 6.7% took traditional/other treatment. In terms of treatment response, 72.44% noticed improvement and 43.1% reported positive treatment effectiveness, whereas 15.9% reported negative.

Table S1 visualizes the exposure and contact history of the participants that portrays 11.7% had scabies before COVID-19. We have found that most of the participants were infected a single time, 26.1% were infected twice, and 17.5% were infected thrice, and 16.6% at least four times. Family members of 82.81% of all the participants had scabies, whereas 52.72% family members had skin diseases. Besides, 65.62% had itching or rash. Table S2 visualizes the clinical symptoms and diagnosis, such that 65.6% have current itching, 63.04% have visible rash, and 46.7% presented with pus discharge. Overall, 85.10% were prescribed by the doctors. Notably, 44.3% have severe scabies, 44.9% had moderate scabies, and 10.7% had mild scabies.

**Figure 2:**
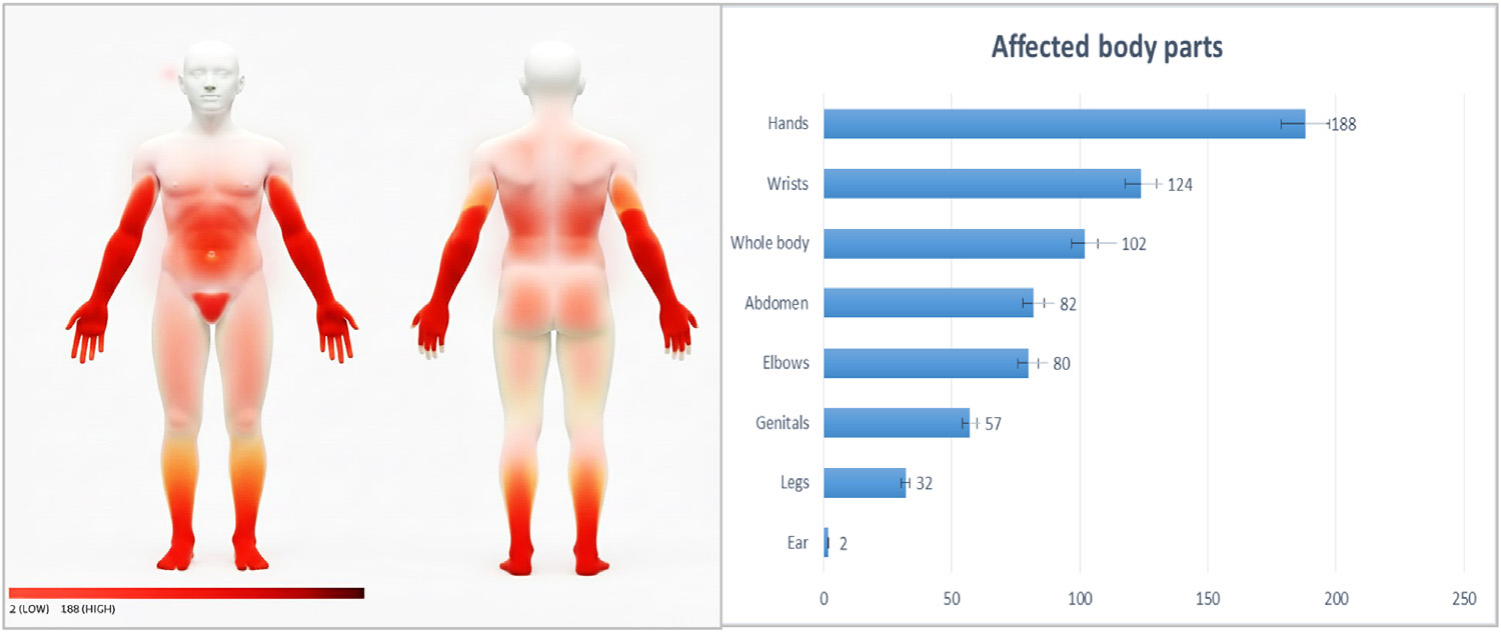
Affected body parts of the respondents.

Figure 2 illustrates the affected body parts among the participants, and the most affected area was the hands, with 188 people affected. The wrists were the next most affected area (n = 124), followed by the whole body (n = 102). Other areas that were affected include the abdomen (n = 82), elbows (n = 80), and genitals (n = 57) among the participants.

### Geographical variation of severity and reinfection rate

**Figure 3:**
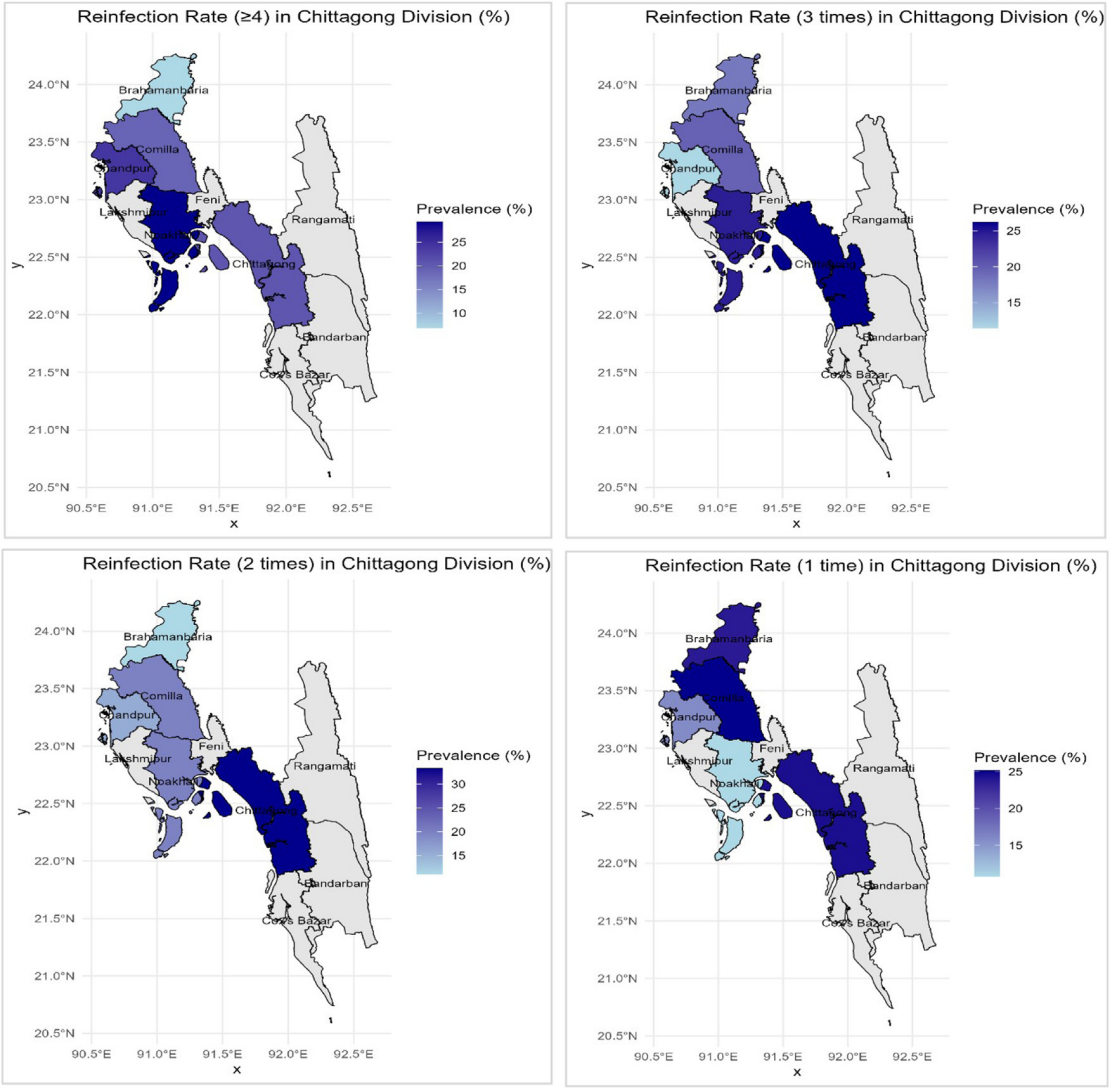
District-wise variation in reinfection rate.

From Figure S5, severe and moderate scabies prevalence is the highest in Chattogram District, indicating the hotspot in the division. Comilla, Bramhanbaria, and Noakhali also show notably higher prevalence for severe scabies. Reinfection of scabies in Chattogram Division shows a strong hotspot pattern, with Chattogram District consistently showing the highest reinfection rates across all categories (one time to four or more times) (Figure 3). Noakhali shows the second highest prevalence in case of three times reinfection rates. All other districts except Chattogram shows lower reinfection rates.

### Bivariate associations

The severity of scabies was significantly associated with several socio-demographic, environmental, and clinical factors (Table S3). Lower educational attainment and older age showed a strong relationship with higher severity because participants with no formal education (64.9%) and older individuals aged ≥51 years (61.9%) had the highest severe cases. Moreover, a severe dust environment was strongly associated with severe scabies (78.7%). Furthermore, poor hygiene maintenance, particularly, non-use of soap, was associated with greater severity (70%). In addition, participants with higher reinfection rates experienced more severe cases (63.8%).

Table S4 found that reinfection rate was significantly associated with educational attainment, bathing frequency, and use of mosquito nets. Participants who do not bathe frequently had a higher proportion of four or more reinfections (27.1%). Moreover, participants with family skin infection (22.3%), current itching (21.4%), and visible rashes (23.2%) and those with no improvement after treatment were significantly associated with four or more reinfections.

In Figure S6, a restricted cubic spline (RCS) curve shows how the severity and reinfection rate are related to age. The RCS curve shows a non-linear association between age and the associated risk of outcome variables, meaning that the relationship between age and risky outcomes does not change in an even manner but, instead, varies for each of the five age groups studied.

Figure S7 shows the adjusted predicted probabilities of scabies severity by reinfection rates. The probability of severe scabies increases exponentially with higher reinfection frequency, whereas the probability of mild disease declines.

Figure S8 reveals that combined treatment (medicine and ointment) had significantly better effectiveness than single treatment (especially the ointment alone). On the other hand, traditional methods were also less effective than combined treatment.

### Factors associated with the severity of scabies

The multinomial logistic regression model reveals a significant association between several factors and the severity of the disease (Table 1). The model shows great discrimination power across all categories (Figure S9). Children had almost 15 times higher severity (RRR = 15.13; 95% confidence interval [CI] 1.83-125.06) than those between the ages of 21 and 30 years, which is about 4.5 times higher in adults aged ≥50 years (RRR = 4.52; 95% CI 1.13-18.04). The severity of scabies was about 15 times more prevalent among participants with no formal education (RRR = 15.69; 95% CI 3.36-73.26), whereas those with only a primary education were 2.83 times more likely to have moderate (RRR = 2.83; 95% CI 1.03-7.77) and 5.34 times more likely to have severe scabies (RRR =5.34; 95% CI 1.88-15.19) than those with a higher education level. Moreover, individuals who did not bathe frequently had a 4.39 times higher risk of moderate scabies (RRR = 4.39; 95% CI 1.00-19.29). Participants with poor hygiene had a 3.5 times higher risk of severe scabies (RRR = 3.5; 95% CI 1.09-11.20) than participants with excellent hygiene. Furthermore, participants with pus discharge from the affected parts have almost 4.21 times higher severity of scabies (RRR = 4.21; 95% CI 1.94-9.14). Moreover, the respondents with four or more times of reinfection are likely to have eight times higher risk of severity (RRR = 8.09; 95% CI 1.78-36.73).

**Table 1:** Factors associated with the severity of scabies.

Characteristics	Moderate		Severe	
	RRR (95% CI)	Significance	RRR (95% CI)	Significance
**Age, years**				
≤10	7.32 (0.90-59.74)	0.063	15.13 (1.83-125.06)	0.012
11-20	1.17 (0.47-2.94)	0.738	1.71 (0.65-4.51)	0.280
21-30	1.00		1.00	
31-40	0.88 (0.28-2.71)	0.821	1.65 (0.52-5.23)	0.393
41-50	0.98 (0.23-4.12)	0.973	2.78 (0.67-11.48)	0.157
≥51	1.27 (0.31-5.20)	0.741	4.52 (1.13-18.04)	0.033
**Gender**	7.32 (0.90-59.74)	0.063		
Female	1.00			
Male	1.15 (0.55-2.39)	0.715	1.50 (0.72-3.13)	0.283
**Education**				
No education	4.00 (0.85-18.72)	0.078	15.69 (3.36-73.26)	<0.001
Primary	2.83 (1.03-7.77)	0.043	5.34 (1.88-15.19)	0.002
Secondary	0.76 (0.31-1.86)	0.544	1.55 (0.61-3.93)	0.361
Higher	1.00			
**Marital status**				
Child	3.29 (1.04-10.47)	0.043	4.50 (1.41-14.35)	0.011
Married	1.24 (0.56-2.74)	0.595	1.69 (0.76-3.75)	0.200
Divorced/widowed/single	1.00		1.00	
**Occupation**				
Agri/small business	1.76 (0.21-14.86)	0.604	4.59 (0.57-37.14)	0.153
Housewife	1.13 (0.46-2.77)	0.795	1.84 (0.75-4.56)	0.185
Job holders	0.13 (0.03-0.60)	0.009	0.79 (0.25-2.51)	0.685
Others	0.88 (0.17-4.46)	0.876	2.79 (0.59-13.13)	0.195
Student	1.00			
**Bathing**				
Frequently				
Not frequently	4.39 (1.00-19.29)	0.050	1.90 (0.42-8.72)	0.408
**Soap use**				
No				
Yes	1.21 (0.24-6.09)	0.817	0.36 (0.08-1.61)	0.180
**Dust exposure**				
Severe	0.57 (0.15-2.16)	0.410	3.00 (0.94-9.60)	0.064
Moderate	1.96 (0.83-4.62)	0.126	1.03 (0.43-2.43)	0.952
Low	1.00			
**Self-reported hygiene**				
Excellent	1.00			
Good	4.13 (1.37-12.45)	0.012	2.78 (0.95-8.11)	0.061
Poor	3.19 (0.96-10.65)	0.059	3.50 (1.09-11.20)	0.035
**Pus discharge**				
No	1.00			
Yes	0.89 (0.41-1.94)	0.775	4.21 (1.94-9.14)	<0.001
**Sought treatment**				
No	1.00			
Yes	2.19 (0.82-5.85)	0.118	3.36 (1.18-9.54)	0.023
**Treatment place**				
Formal	3.32 (1.44-7.65)	0.005	2.89 (1.27-6.59)	0.012
Informal	1.00			
**Treatment type**				
Medicine	5.03 (1.16-21.85)	0.031	4.50 (1.00-20.31)	0.050
Ointment	2.77 (0.83-9.24)	0.097	3.08 (0.90-10.59)	0.074
Ointment and medicine	6.39 (1.90-21.43)	0.003	7.80 (2.26-26.88)	<0.001
Traditional method	1.00			
**Reinfection times**				
1	1.00			
2	1.24 (0.55-2.79)	0.599	1.09 (0.47-2.54)	0.835
3	8.81 (1.13-68.93)	0.038	14.87 (1.91-115.96)	0.010
≥4	3.22 (0.69-14.99)	0.137	8.09 (1.78-36.73)	0.007

CI, confidence interval; RRR, Relative Risk Ratio.

### Factors associated with reinfection rate of scabies

Table 2 presents a detailed results of factors associated with reinfection rate. Model participants with ≤10 years (RRR = 4.64; 95% CI 1.57-13.74) and 11-20 years age group (RRR = 4.31; 95% CI 1.61-11.51) had more than four times higher risk of experiencing four or more reinfections, whereas the elderly group aged ≥51 years had more than three times higher risk than those aged 21-30 years (RRR = 3.46; 95% CI 1.15-10.43). Respondents with low educational level, such as with primary education, had significantly higher risk of reinfection rates than those with higher education. Notably, the type of residence is another important determinant. Individuals living in tin sheds faced a significantly higher reinfection rate, with 2.61 times increased risk for two occurrences (RRR = 2.61; 95% CI 1.34-5.13) and 2.79 times increased risk for four or more occurrences (RRR = 2.79; 95% CI 1.33-5.88). Hygiene practices, particularly, frequency of bathing, strongly correlate with reinfection rates. Individuals who did not bathe frequently showed highly significant likelihood of experiencing reinfection of scabies.

**Table 2:** Factors associated with the reinfection rate of scabies.

Characteristics	Two times		Three times		Four times or more	
	RRR (95% CI)	Significance	RRR (95% CI)	Significance	RRR (95% CI)	Significance
**Age, years**						
≤10	2.09 (0.9-4.86)	0.086	2.03 (0.73-5.67)	0.177	4.64 (1.57-13.74)	0.006
11-20	1.8 (0.86-3.77)	0.118	2.38 (1.00-5.66)	0.050	4.31 (1.61-11.51)	0.004
21-30	1.00					
31-40	2.09 (0.83-5.25)	0.117	2.95 (1.04-8.35)	0.042	3.54 (1.05-11.93)	0.042
41-50	1.12 (0.41-3.02)	0.830	1.28 (0.39-4.22)	0.688	1.31 (0.3-5.73)	0.716
≥51	0.66 (0.23-1.89)	0.438	1.41 (0.48-4.14)	0.529	3.46 (1.15-10.43)	0.028
**Education level**						
No education	0.93 (0.42-2.05)	0.856	2.07 (0.87-4.94)	0.102	2.05 (0.88-4.79)	0.098
Primary	2.52 (1.29-4.93)	0.007	2.90 (1.27-6.63)	0.012	2.31 (1.05-5.30)	0.049
Secondary	0.80 (0.35-1.81)	0.586	1.66(0.67-4.1)	0.276	1.28 (0.51-3.25)	0.602
Higher	1.00					
**Occupation**						
Housewife	1.00					
Agri/business	1.54 (0.48-4.93)	0.469	0.82 (0.23-2.94)	0.761	0.68 (0.17-2.78)	0.595
Job holders	1.92 (0.58-6.42)	0.288	1.03 (0.28-3.81)	0.971	0.85 (0.2-3.6)	0.830
Others	2.32 (0.83-6.55)	0.109	0.37 (0.08-1.84)	0.226	1.03 (0.31-3.42)	0.955
Student	1.96 (1-3.88)	0.051	0.92 (0.47-1.82)	0.813	0.95 (0.47-1.94)	0.903
**Household size**						
1-3	1.00					
4-6	1.07(0.4-2.81)	0.898	3.3(0.7-15.53)	0.131	2.82(0.6-13.32)	0.191
≥7	0.6(0.21-1.71)	0.341	1.26(0.24-6.52)	0.782	1.72(0.34-8.64)	0.511
**Residence types**						
Building	1.00					
Mud house	1.39 (0.53-3.65)	0.503	1.15 (0.38-3.51)	0.803	0.8 (0.21-3.04)	0.747
Tin shed	2.61 (1.34-5.13)	0.005	1.73 (0.79-3.82)	0.171	2.79 (1.33-5.88)	0.007
**Mosquito net**						
Yes	1.00					
No	2.12 (1.22-3.68)	0.008	1.6 (0.85-3.02)	0.142	0.72 (0.35-1.47)	0.360
**Bathing**						
Frequently						
Not frequently	4.64 (1.86-11.65)	<0.001	3.69 (1.34-10.24)	0.012	5.44 (2.05-14.5)	<0.001
**Self-reported hygiene**						
Good	2.44 (0.95-6.3)	0.064	1.12 (0.33-3.87)	0.854	1.33 (0.38-4.63)	0.650
Excellent	1.88 (1.04-3.41)	0.037	1.41 (0.72-2.77)	0.317	1.94 (0.99-3.79)	0.052
Poor	1.00					
**Sought treatment**						
No	1.00					
Yes	6.61 (1.5-29.26)	0.013	1.14 (0.45-2.91)	0.772	1.57 (0.56-4.47)	0.392
**Treatment place**						
Formal	1.83(0.94-3.55)	0.076	3.33(1.31-8.46)	0.011	1.72(0.78-3.79)	0.178
Informal	1.00					
**Treatment type**						
Ointment	1.75 (0.79-3.9)	0.171	1.98 (0.69-5.71)	0.206	0.82 (0.2-3.31)	0.777
Medicine	1.26 (0.68-2.35)	0.460	2.72 (1.24-5.96)	0.012	3.18 (1.42-7.08)	0.005
Both	0.87 (0.24-3.17)	0.839	2.78 (0.76-10.16)	0.121	3.06 (0.83-11.33)	0.094
Traditional	1.00					
**Family skin infection**						
No	1.00					
Yes	1.44 (0.85-2.46)	0.171	1.37 (0.75-2.51)	0.306	2.99 (1.55-5.78)	<0.001
**Currently itching**						
No	1.00					
Yes	2.14 (1.22-3.75)	0.008	2.15 (1.14-4.11)	0.019	4.92 (2.25-10.79)	<0.001
**Visible rash**						
No	1.00					
Yes	1.58 (0.92-2.73)	0.094	1.49 (0.81-2.76)	0.201	6.58 (2.79-15.52)	<0.001
**Improvement after treatment**						
Yes	1.00					
No	2.5 (1.22-5.16)	0.013	2.99 (1.36-6.57)	0.006	10.13 (4.67-22)	<0.001
**Effectiveness of the treatment**						
Yes	1.00					
No	5.03 (1.75-14.45)	0.003	5.21 (1.71-15.92)	0.004	22 (7.4-65.44)	<0.001

CI, confidence interval; RRR, Relative Risk Ratio.

In terms of treatment-seeking behavior and treatment-seeking place, those who sought treatment had a significantly higher likelihood of being reinfected two times (RRR = 1.83; 95% CI 0.94-3.55). Household transmission is another significant factor, with participants reporting family members with skin infections being almost three times more likely (RRR = 2.99; 95% CI 1.55-5.78) to experience four or more reinfections. In addition, current and visible rashes are significantly associated with higher reinfection rates. Receiver-operating characteristic curves were calculated using area under the curve (AUC) for each category, using a one-vs-rest approach, to assess the ability of the model to discrimination power. The AUC for all categories were greater than 80%, indicating good performance of the model (Figure S9).

## Discussion

This study reveals that the current situation of scabies in Bangladesh is progressing from a dermatological condition to more complicated forms. Our study found that 44.35% of respondents were experiencing severe levels of scabies, with almost 34.1% of participants having reinfection of three or more times. This severity and reinfection rates are likely to be exacerbated by a combination of environment, transmission, living conditions, and hygiene maintenance [[16], [17], [18]].

A core explanation for this high burden of severe scabies is may be due to late diagnosis and long-term infestation [19]. Delayed treatment of scabies may lead to increased mite burden, more extensive lesions, and disruption of the skin due to constant scratching. This increases secondary bacterial infection, which can deteriorate the situation and may contribute to complicated scabies infection [20]. Scabies is a more common infectious disease than a purely dermatological condition, as reported by evidence, because secondary infections increase the risk of more serious conditions such as rheumatic heart disease and post-streptococcal glomerulonephritis, in addition to contributing to immediate morbidity [21,22].

The reinfection rates found in our study match the well-established, accepted concept that scabies is mostly a household disease. Close contact, sharing sleeping spaces, bedding items, and unhygienic living circumstances are the primary causes of transmission [23]. Based on research conducted on Cox’s Bazar Rohingya camps and madrasah students in Dhaka, close physical contact, limited space, and shared living environments are all significantly associated with the spread of scabies [24]. Finally, the regional hotspot nature of scabies in Chattogram Division is likely due to population density and socio-environmental inequalities. Therefore, in addition to ineffective scabies treatments, the Chattogram Division’s reinfection pressure could be ascribed, in part, to a lack of planned household scabies treatment and insufficient environmental hygiene.

Educational attainment is the key of severity and reinfection. This relationship is mediated by health literacy, treatment, and prevention knowledge. Participants with lower levels of education may have a poor understanding of how scabies spreads and the treatment process [25]. They may also postpone seeking medical treatment until symptoms worsen. Similar results have been found in the existing literature [26]. Age also implies higher prevalence and reinfection across all age groups, especially children, who have a greater infection prevalence. This is may be due to frequent close contact in schools, late diagnosis, and poor personal hygiene practices. Weak immunity, comorbidities, or a lack of personal and catastrophic health expenditures may raise the risk of severe scabies infection in older cohort [27]. The finding aligns with the previous literature in Ethiopia [[28], [29], [30]], Uganda [31], Australia [32], and others [4,33]. Hygiene factors such as regular bathing and soap use have a significant association with scabies prevalence. Poor hygiene and insufficient use of soap and clean water might lead to long-term infestation. In densely crowded regions, poor hygiene becomes more difficult because mites can stick to clothing, shared items, and beds, increasing the risk of transmission even after treatment [22,33].

Environmental factors, such as excessive dust, may be related to scabies severity. It can irritate the skin, stimulate scratching, and intensify the problem. Household transmission was found as a major factor of scabies in this regard because many respondents had infected family members, and family skin infection raised the likelihood of reinfection drastically. This supports existing evidence [34]. Reinfection is common when only the symptomatic person is treated, leaving other household members untreated, or when bedding and clothing are not properly cleaned. Scabies transmission through the house was another key factor in our study; there was a significant association between skin infection in family members and the likelihood of reinfection. This finding is consistent with previous research [35,36].

According to the multivariable analysis, scabies severity and reinfection tend to be associated with socio-demographic condition, poor hygiene maintenance, and ongoing household transmission [37]. Children and the elderly individuals are more likely to develop severe scabies [38]. Lower education was consistently linked with greater severity and reinfection, indicating limited health awareness and inadequate preventive practices. Similar results have been found in the existing literature [39]. Irregular bathing and poor hygiene were significant predictors of both outcomes. People who lived in tin sheds or with infected family members were at a higher risk of reinfection [40]. Furthermore, reinfection frequency and infection severity have a strong positive relationship. This could point to a continued cycle of reinfection and an inability to completely remove the sickness.

This study emphasizes that rather than treating scabies symptoms alone, household-focused and community-based treatments are needed to control the disease in Bangladesh. Healthy lifestyle knowledge may be effective in case of handing these types diseases [41,42]. Furthermore, 79.01% of the participants who sought treatment received formal treatment. In the context of Bangladesh, formal treatment typically involves prescribing treatment for affected household members or close contacts. Therefore, it is likely that household-level treatment was generally recommended in the study setting.

Our findings indicate that individuals who have had severe scabies infection are at a much higher risk of reinfection. Therefore, more intensive and prolonged treatment regimens may have to be used, especially for those who have had severe scabies. In severe cases, such as hyperkeratotic scabies, additional supportive measures, such as removal of thick crusts and proper nail care, are necessary to increase the effectiveness of treatment [43]. For common scabies, the first-line therapy is topical permethrin or phenothrin or oral ivermectin. Second-line therapy for scabies includes sulfur, crotamiton cream, and benzyl benzoate [44,45].

## Conclusion

This study reveals a significant burden of scabies severity, reinfection, and treatment effectiveness in the Chattogram Division of Bangladesh. A larger portion of participants experienced moderate to severe scabies, with most facing three or more reinfections within the last 12 months. These findings indicate that scabies is not only a dermatological disease but also a significant and emerging community-level public health issue. Mostly, children and older adults were more likely to experience severe conditions. Lower education, poor hygiene practices, households, and environmental factors, such as a family history of scabies, are strongly and significantly associated with higher severity and reinfection rates. In terms of treatment response, a combined approach using medication and ointment is more effective than using either a single method or traditional treatment alone. Although Bangladesh’s medical sector is highly subsidized, it is still significantly lacking in controlling these types of communicable diseases. These findings emphasize the need for a control strategy for handling scabies, especially in rural areas. Moreover, community-based awareness programs, maintaining good hygiene, early diagnosis, and treatment targeting infected persons and family members are essential to reduce this severity level and reinfection rates of scabies.

### Strengths and limitations

One of the study’s many advantages is the multi-district coverage in the Chattogram Division, with multi-stage clustered sampling and comprehensive assessment of socio-demographic, clinical, hygiene, environmental, and treatment-related factors. Moreover, the study was conducted during recent surges of scabies outbreaks, which provide the real scenarios of the current situation. However, cross-sectional designs limit the causal relationship, and primarily collected data may produce recall and reporting bias. Information on simultaneous treatment and the exact number of affected household members was not collected, limiting detailed analysis of household transmission.

### Policy recommendations

To control scabies in the Chattogram area, community programs that emphasize early identification and prompt treatment must be created. All household members and those they often interact should receive treatment simultaneously. A hygiene message that emphasizes frequent bathing, soap use, and bed and clothes laundering must be spread throughout homes in addition to treatment.

## Declaration of competing interest

We declare no competing interests. During the preparation of this work, the authors used ChatGPT solely for language and style editing assistance. After using this tool, the authors thoroughly reviewed and revised the content and took full responsibility for the accuracy and integrity of the publication.

## Appendix Supplementary materials

Image, application 1
