# Supplementary material for: Factors associated with scabies severity and reinfection: A cross-sectional study during recent surges in the Chattogram Division, Bangladesh
Source: IJID Reg. 2026 Apr 15;19:100899. doi: 10.1016/j.ijregi.2026.100899 (PMC13147370; doi:10.1016/j.ijregi.2026.100899)
Supplement: Supplementary file 1 [file mmc1.docx]

**Supplementary Materials**

**Table S1: Exposure and contact history of the participants**

| **Characteristics** | **Categories** | **n (%)** |
| --- | --- | --- |
| Scabies before COVID | No | 308 (88.25) |
|  | Yes | 41 (11.75) |
| Reinfection | 1 | 139 (39.83) |
|  | 2 | 91 (26.07) |
|  | 3 | 61 (17.48) |
|  | ≥ 4 | 58 (16.62) |
| Family members had scabies | No | 60 (17.19) |
|  | Yes | 289 (82.81) |
| Family members had skin diseases | No | 165 (47.28) |
|  | Yes | 184 (52.72) |
| Someone with itching or rash | No | 120 (34.38) |
|  | Yes | 229 (65.62) |

**Table S2: Clinical symptoms and diagnosis related characteristics of the participants**

| **Characteristics** | **Categories** | **n (%)** |
| --- | --- | --- |
| Current itching | No | 120 (34.38) |
|  | Yes | 229 (65.62) |
| Visible rash | No | 129 (36.96) |
|  | Yes | 220 (63.04) |
| Pus discharge | No | 179 (53.27) |
|  | Yes | 157 (46.73) |
| Doctor diagnosed | No | 52 (14.90) |
|  | Yes | 297 (85.10) |
| Severity | Crusted/ severe | 149 (44.35) |
|  | Moderate | 151 (44.94) |
|  | Mild | 36 (10.71) |

**Table S3: Association between Severity and its potential factors**

| **Characteristics** | **Severity** | | | **P-value** |
| --- | --- | --- | --- | --- |
|  | **Crusted** | **Moderate** | **Mild** |  |
| **Age** |  |  |  | **0.033** |
| ≤10 | 29 (52.7) | 25 (45.5) | 1 (1.8) |  |
| 11–20 | 36 (39.6) | 44 (48.4) | 11 (12.1) |  |
| 21–30 | 23 (30.3) | 41 (53.9) | 12 (15.8) |  |
| 31–40 | 19 (44.2) | 18 (41.9) | 6 (14.0) |  |
| 41–50 | 16 (55.2) | 10 (34.5) | 3 (10.3) |  |
| ≥51 | 26 (61.9) | 13 (31.0) | 3 (7.1) |  |
| **Gender** |  |  |  |  |
| Female | 72 (40.9) | 83 (47.2) | 21 (11.9) | 0.387 |
| Male | 77 (48.1) | 68 (42.5) | 15 (9.4) |  |
| **Education** |  |  |  |  |
| No Education | 48 (64.9) | 24 (32.4) | 2 (2.7) | **<0.001** |
| Primary | 49 (46.2) | 51 (48.1) | 6 (5.7) |  |
| Secondary | 26 (41.9) | 25 (40.3) | 11 (17.7) |  |
| Higher | 26 (27.7) | 51 (54.3) | 17 (18.1) |  |
| **Marital Status** |  |  |  |  |
| Child | 45 (50.0) | 41 (45.6) | 4 (4.4) | 0.104 |
| Married | 45 (37.8) | 56 (47.1) | 18 (15.1) |  |
| Divorced/Widowed/Single | 59 (46.5) | 54 (42.5) | 14 (11.0) |  |
| **Occupation** |  |  |  |  |
| Agri/Small Business | 14 (60.9) | 8 (34.8) | 1 (4.3) | **0.006** |
| Housewife | 45 (47.9) | 41 (43.6) | 8 (8.5) |  |
| Job Holders | 12 (60.0) | 3 (15.0) | 5 (25.0) |  |
| Others | 17 (63.0) | 8 (29.6) | 2 (7.4) |  |
| Student | 61 (35.5) | 91 (52.9) | 20 (11.6) |  |
| **Bathing** |  |  |  |  |
| Frequently | 134 (46.5) | 120 (41.7) | 34 (11.8) | **0.010** |
| Not Frequently | 15 (31.3) | 31 (64.6) | 2 (4.2) |  |
| **Soap Use** |  |  |  |  |
| No | 21 (70.0) | 7 (23.3) | 2 (6.7) | **0.012** |
| Yes | 128 (41.8) | 144 (47.1) | 34 (11.1) |  |
| **Dust Exposure** |  |  |  |  |
| Severe | 48 (78.7) | 8 (13.1) | 5 (8.2) | **<0.001** |
| Moderate | 69 (33.7) | 115 (56.1) | 21 (10.2) |  |
| Low | 32 (45.7) | 28 (40.0) | 10 (14.3) |  |
| **Self-reported Hygiene** |  |  |  |  |
| Excellent | 11 (40.7) | 9 (33.3) | 7 (25.9) | **0.025** |
| Good | 83 (40.9) | 101 (49.8) | 19 (9.4) |  |
| Poor | 55 (51.9) | 41 (38.7) | 10 (9.4) |  |
| **Pus Discharge** |  |  |  |  |
| No | 47 (27.0) | 103 (59.2) | 24 (13.8) | **<0.001** |
| Yes | 99 (63.1) | 46 (29.3) | 12 (7.6) |  |
| **Sought Treatment** |  |  |  |  |
| No | 10 (31.3) | 15 (46.9) | 7 (21.9) | 0.064 |
| Yes | 139 (45.7) | 136 (44.7) | 29 (9.5) |  |
| **Treatment Place** |  |  |  |  |
| Formal | 112 (45.7) | 115 (46.9) | 18 (7.3) | **0.011** |
| Informal | 28 (42.4) | 25 (37.9) | 13 (19.7) |  |
| **Treatment Type** |  |  |  |  |
| Medicine | 18 (40.0) | 23 (51.1) | 4 (8.9) | **0.015** |
| Ointment | 37 (42.5) | 38 (43.7) | 12 (13.8) |  |
| Ointment & Medicine | 78 (48.4) | 73 (45.3) | 10 (6.2) |  |
| Traditional Method | 7 (31.8) | 8 (36.4) | 7 (31.8) |  |
| **Reinfection** |  |  |  |  |
| 1 | 48 (36.6) | 62 (47.3) | 21 (16.0) | **<0.001** |
| 2 | 30 (34.9) | 44 (51.2) | 12 (14.0) |  |
| 3 | 34 (55.7) | 26 (42.6) | 1 (1.6) |  |
| ≥4 | 37 (63.8) | 19 (32.8) | 2 (3.4) |  |

**Table S4: Association between reinfection and its potential factors**

| **Characteristics** | **Reinfection** | | | | **P-value** |
| --- | --- | --- | --- | --- | --- |
|  | **1 time** | **2 times** | **3 times** | ≥**4** |  |
| **Age** |  |  |  |  |  |
| ≤10 | 17 (30.9) | 17 (30.9) | 9 (16.4) | 12 (21.8) | 0.135 |
| 11–20 | 29 (31.9) | 25 (27.5) | 18 (19.8) | 19 (20.9) |  |
| 21 –30 | 46 (52.9) | 22 (25.3) | 12 (13.8) | 7 (8.0) |  |
| 31–40 | 13 (30.2) | 13 (30.2) | 10 (23.3) | 7 (16.3) |  |
| 41–50 | 15 (48.4) | 8 (25.8) | 5 (16.1) | 3 (9.7) |  |
| ≥51 | 19 (45.2) | 6 (14.3) | 7 (16.7) | 10 (23.8) |  |
| **Education level** |  |  |  |  |  |
| No Education | 29 (39.2) | 14 (18.9) | 15 (20.3) | 16 (21.6) | **0.037** |
| Primary | 29 (27.4) | 38 (35.8) | 21 (19.8) | 18 (17.0) |  |
| Secondary | 29 (46.0) | 12 (19.0) | 12 (19.0) | 10 (15.9) |  |
| Higher | 52 (49.1) | 27 (25.5) | 13 (12.3) | 14 (13.2) |  |
| **Occupation** |  |  |  |  |  |
| Housewife | 10 (43.5) | 6 (26.1) | 4 (17.4) | 3 (13.0) | 0.756 |
| Agri/ Business | 41 (43.2) | 16 (16.8) | 20 (21.1) | 18 (18.9) |  |
| Job Holders | 8 (38.1) | 6 (28.6) | 4 (19.0) | 3 (14.3) |  |
| Others | 11 (39.3) | 10 (35.7) | 2 (7.1) | 5 (17.9) |  |
| Student | 69 (37.9) | 53 (29.1) | 31 (17.0) | 29 (15.9) |  |
| **Household Size** |  |  |  |  |  |
| 1-3 | 11 (47.8) | 8 (34.8) | 2 (8.7) | 2 (8.7) | 0.077 |
| 4-6 | 80 (34.6) | 62 (26.8) | 48 (20.8) | 41 (17.7) |  |
| ≥7 | 48 (50.5) | 21 (22.1) | 11 (11.6) | 15 (15.8) |  |
| **Residence types** |  |  |  |  |  |
| Building | 109 (44.3) | 57 (23.2) | 43 (17.5) | 37 (15.0) | 0.075 |
| Mud house | 11 (40.7) | 8 (29.6) | 5 (18.5) | 3 (11.1) |  |
| Tin shed | 19 (25.0) | 26 (34.2) | 13 (17.1) | 18 (23.7) |  |
| **Mosquito Net** |  |  |  |  |  |
| Yes | 99 (43.0) | 49 (21.3) | 37 (16.1) | 45 (19.6) | **0.008** |
| No | 40 (33.6) | 42 (35.3) | 24 (20.2) | 13 (10.9) |  |
| **Bathing** |  |  |  |  |  |
| Frequently | 132 (43.9) | 73 (24.3) | 51 (16.9) | 45 (15.0) | **<0.001** |
| Not Frequently | 7 (14.6) | 18 (37.5) | 10 (20.8) | 13 (27.1) |  |
| **Self-reported Hygiene** |  |  |  |  |  |
| Good | 9 (32.1) | 11 (39.3) | 4 (14.3) | 4 (14.3) | 0.190 |
| Excellent | 96 (44.9) | 48 (22.4) | 38 (17.8) | 32 (15.0) |  |
| Poor | 34 (31.8) | 32 (29.9) | 19 (17.8) | 22 (20.6) |  |
| **Sought Treatment** |  |  |  |  |  |
| No | 18 (56.3) | 2 (6.3) | 7 (21.9) | 5 (15.6) | **0.044** |
| Yes | 121 (38.2) | 89 (28.1) | 54 (17.0) | 53 (16.7) |  |
| **Treatment Place** |  |  |  |  |  |
| Formal | 90 (35.2) | 73 (28.5) | 50 (19.5) | 43 (16.8) | **0.036** |
| Informal | 36 (52.9) | 16 (23.5) | 6 (8.8) | 10 (14.7) |  |
| **Treatment Type** |  |  |  |  |  |
| Ointment | 18 (38.3) | 18 (38.3) | 8 (17.0) | 3 (6.4) | **0.020** |
| Medicine | 49 (50.0) | 28 (28.6) | 11 (11.2) | 10 (10.2) |  |
| Both | 54 (33.5) | 39 (24.2) | 33 (20.5) | 35 (21.7) |  |
| Traditional | 8 (36.4) | 4 (18.2) | 5 (22.7) | 5 (22.7) |  |
| **Family Skin Infection** |  |  |  |  |  |
| No | 77 (46.7) | 42 (25.5) | 29 (17.6) | 17 (10.3) | **0.011** |
| Yes | 62 (33.7) | 49 (26.6) | 32 (17.4) | 41 (22.3) |  |
| **Currently Itching** |  |  |  |  |  |
| No | 66 (55.0) | 27 (22.5) | 18 (15.0) | 9 (7.5) | **<0.001** |
| Yes | 73 (31.9) | 64 (27.9) | 43 (18.8) | 49 (21.4) |  |
| **Visible Rash** |  |  |  |  |  |
| No | 66 (51.2) | 33 (25.6) | 23 (17.8) | 7 (5.4) | **<0.001** |
| Yes | 73 (33.2) | 58 (26.4) | 38 (17.3) | 51 (23.2) |  |
| **Improvement after treatment** |  |  |  |  |  |
| Yes | 103 (45.6) | 63 (27.9) | 39 (17.3) | 21 (9.3) | **<0.001** |
| No | 15 (17.4) | 23 (26.7) | 17 (19.8) | 31 (36.0) |  |
| **Effectiveness of the treatment** |  |  |  |  |  |
| Yes | 72 (52.2) | 31 (22.5) | 23 (16.7) | 12 (8.7) | **<0.001** |
| No | 52 (28.6) | 56 (30.8) | 33 (18.1) | 41 (22.5) |  |


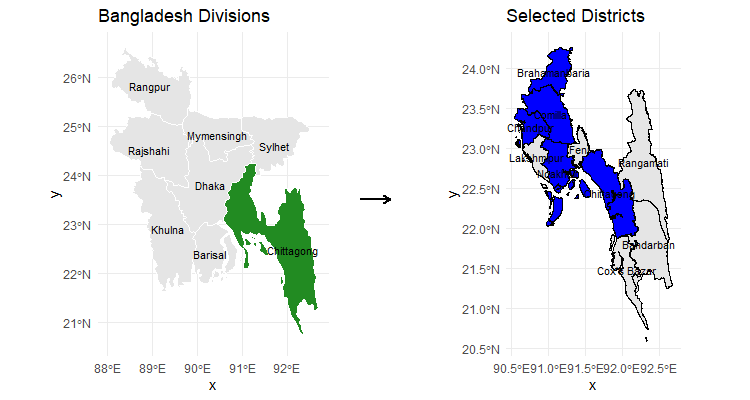


**Fig. S1: Study area map**

**
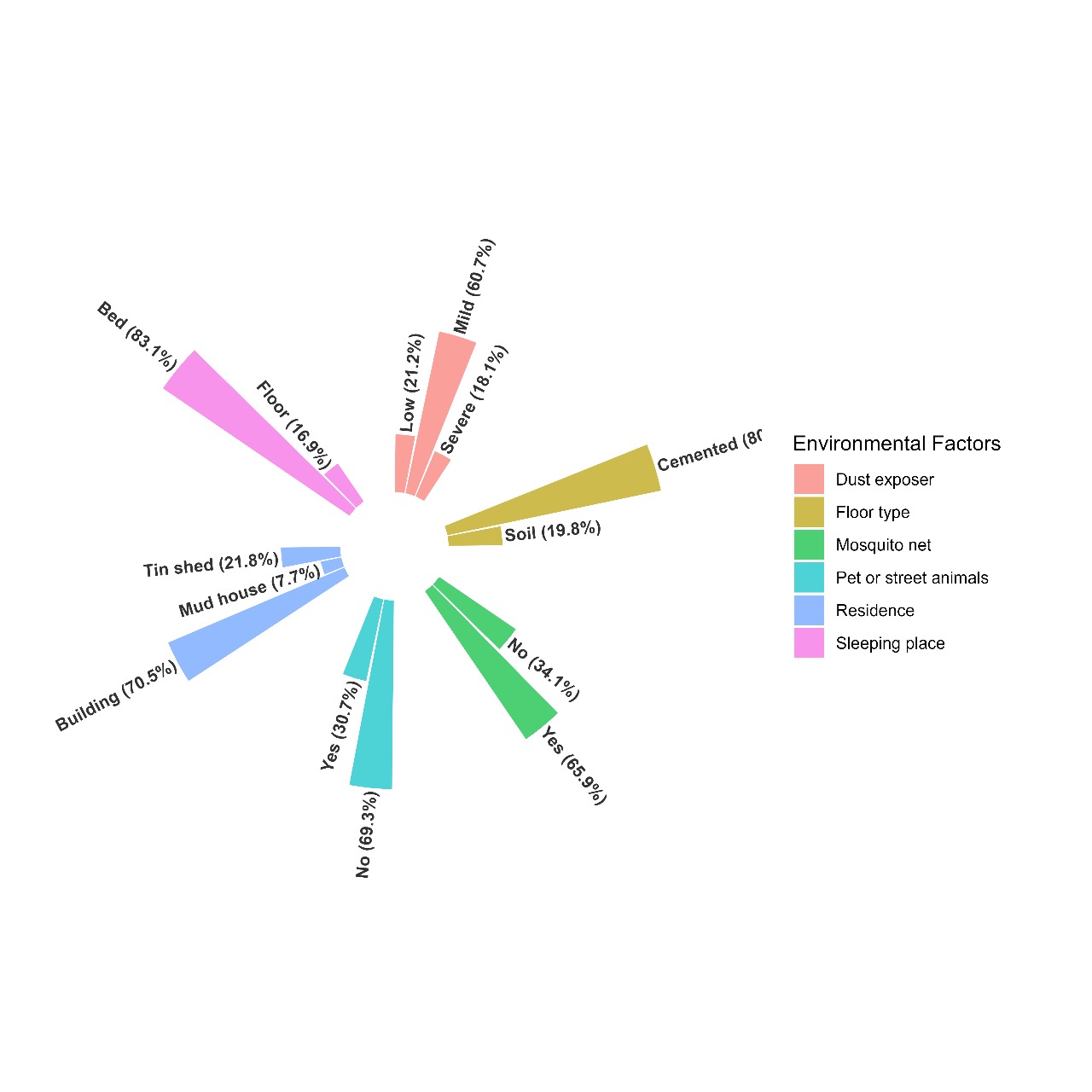
**

**Fig. S2: The distribution of environmental factors among the participants**

**
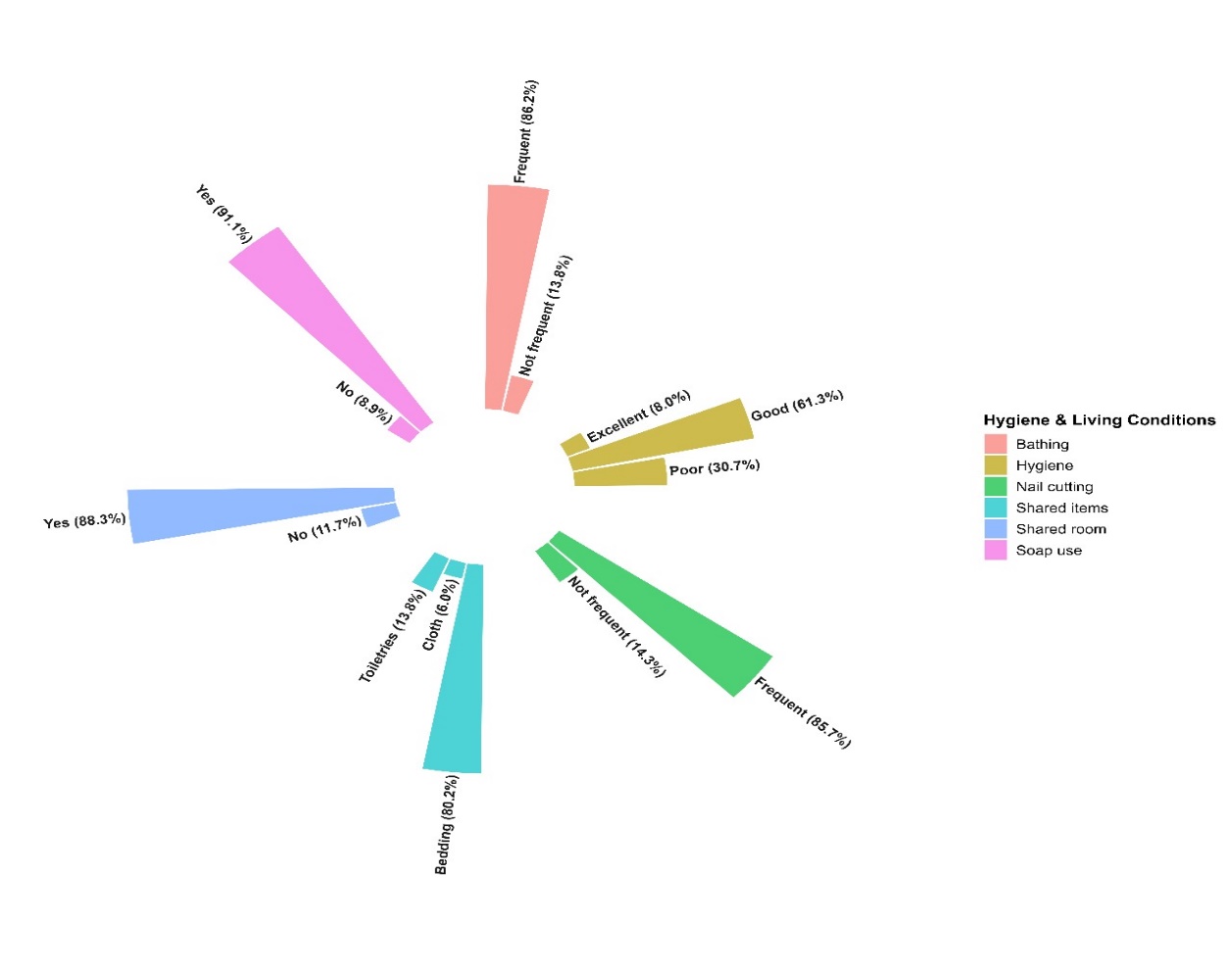
**

Fig. S3**: The hygiene and living conditions of the participants**

**
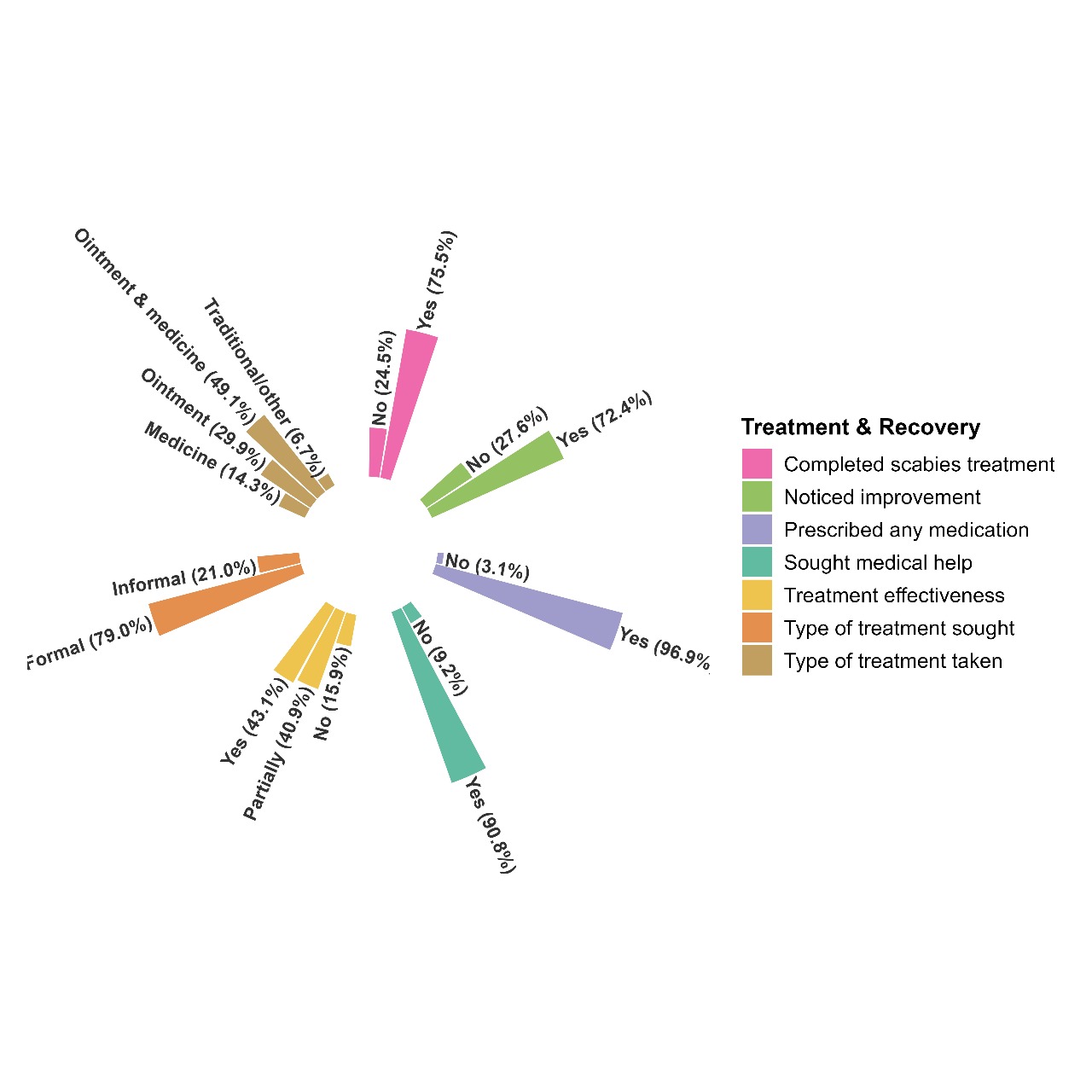
**

**Fig. S4: The treatment and recovery status of the participants**


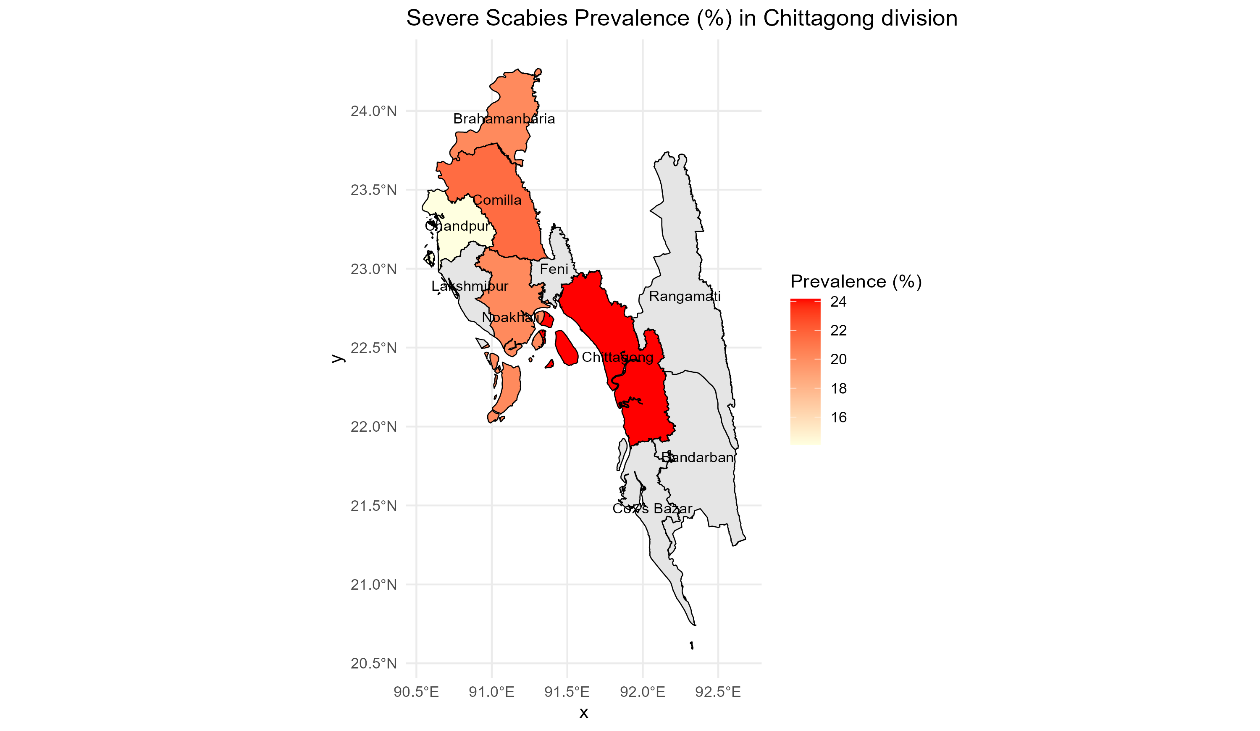

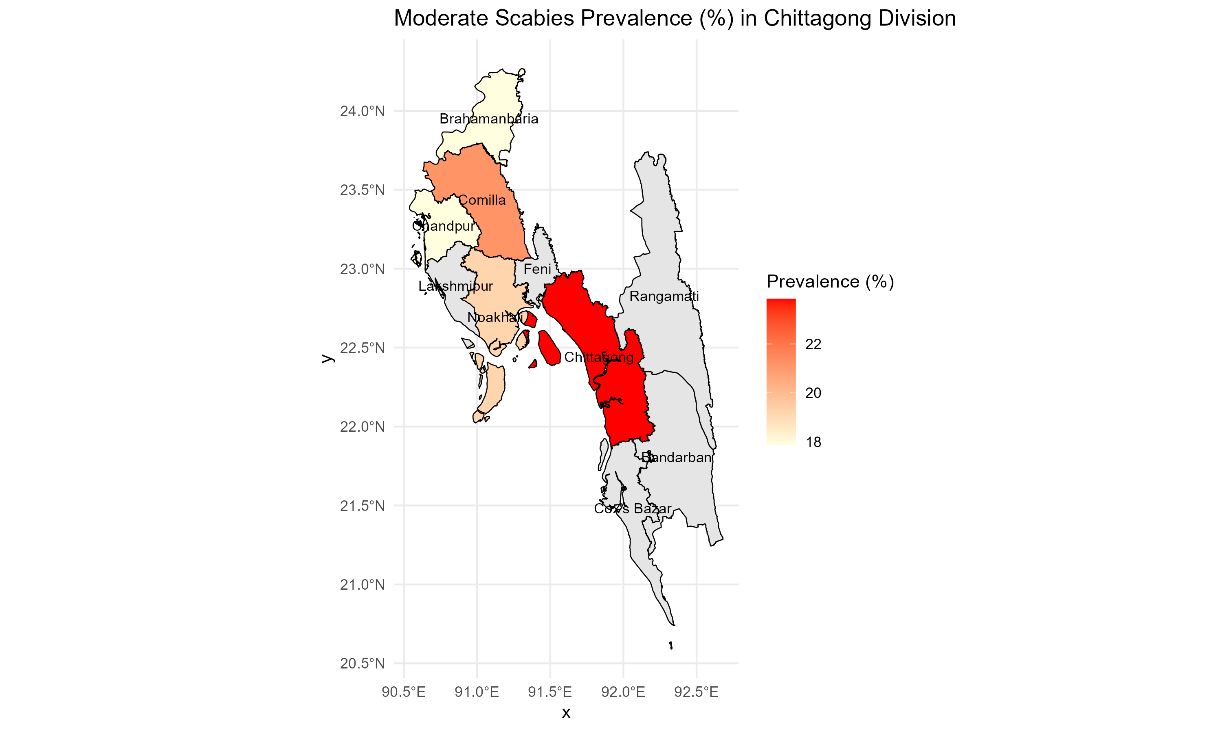


**Fig. S5: District wise variation in prevalence of scabies**


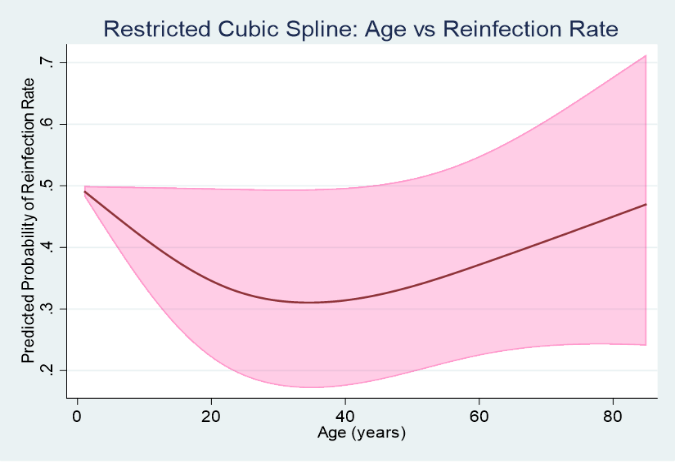


**Fig. S6: RCS Curve between age and outcome variables**

**Fig. S7: Adjusted predicted probabilities of severity by reinfection**

**
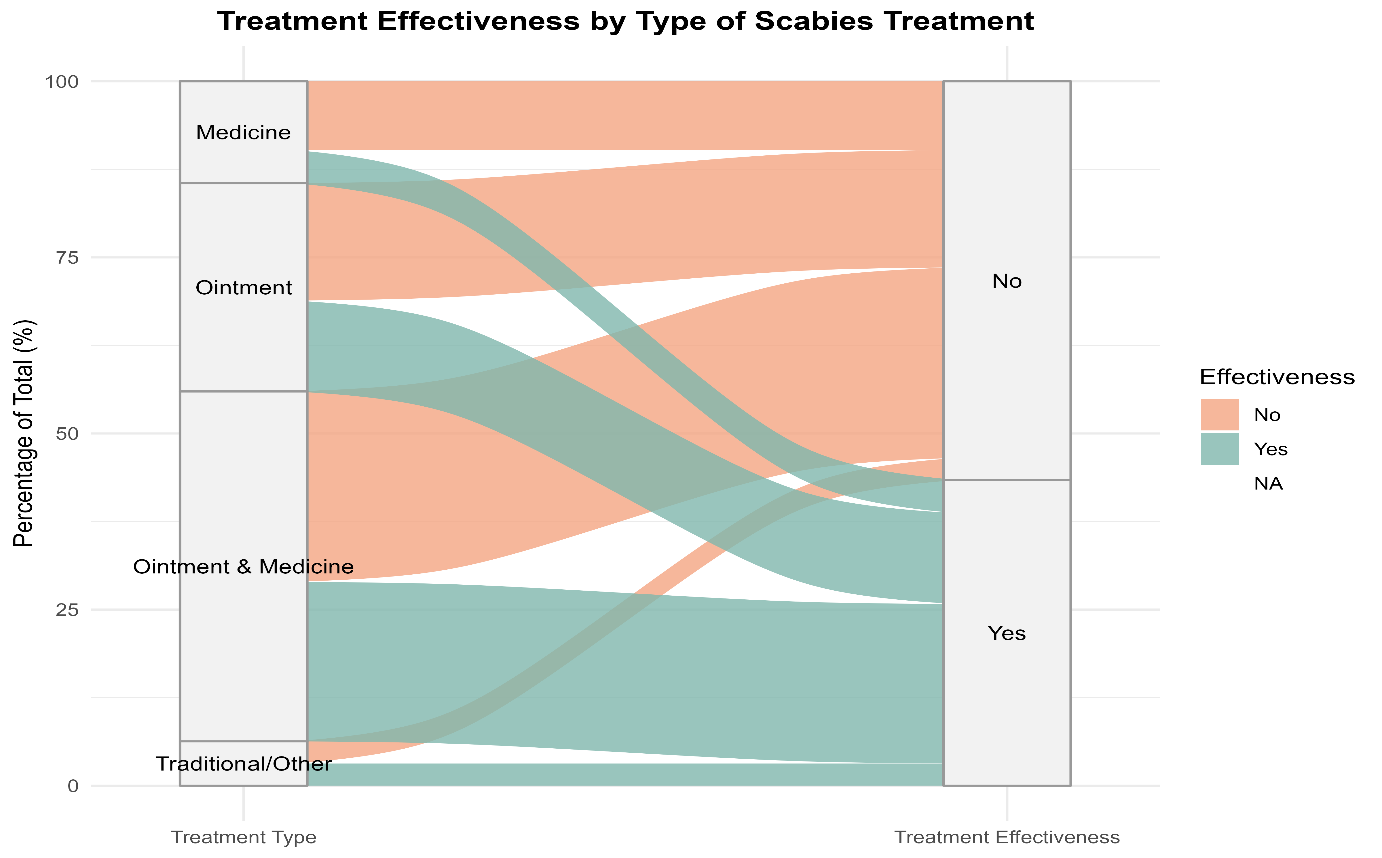
**

**Fig. S8: Treatment effectiveness and treatment types**

**
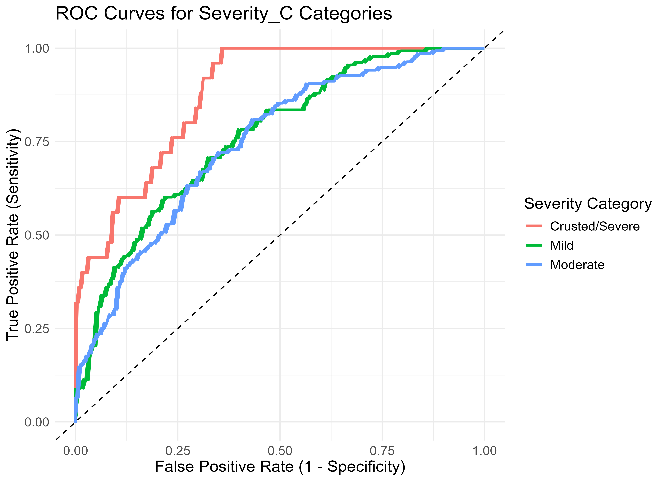

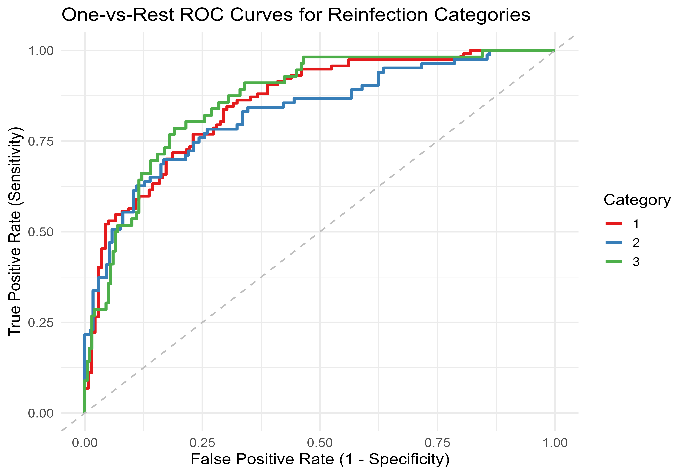
**

**Fig. S9: ROC Curve for model discrimination power**
